# Docosahexaenoic Acid Protects Traumatic Brain Injury by Regulating NOX_2_ Generation via Nrf2 Signaling Pathway

**DOI:** 10.1007/s11064-020-03078-z

**Published:** 2020-07-16

**Authors:** Wei Zhu, Guangqiang Cui, Tuo Li, Hongguang Chen, Jian Zhu, Yuexia Ding, Li Zhao

**Affiliations:** 1 Department of Neurosurgery, The Affiliated Yantai Yuhuangding Hospital of Qingdao University, Yantai, Shandong China; 2 Department of Medical Engineering, The Affiliated Yantai Yuhuangding Hospital of Qingdao University, Yantai, Shandong China; 3 Department of Pharmacy, The Affiliated Yantai Yuhuangding Hospital of Qingdao University, No. 20, East Yuhuangding Road, Zhifu District, Yantai, 264000 Shandong China

**Keywords:** Traumatic brain injury, Docosahexaenoic acid, NADPH oxidase, Nuclear factor erythroid 2-related factor 2, Reactive oxygen species

## Abstract

Docosahexaenoic acid (DHA) is verified to have neuroprotective effects on traumatic brain injury (TBI) rats by activating Nrf2 signaling pathway, but the role of NOX_2_ in this effect has not been illuminated. So this study explored the role of NOX_2_ in TBI models treated with DHA, aiming to complete the mechanism of DHA. TBI rat models were constructed with or without DHA treatment, and H_2_O_2_-induced hippocampal neurons were pretreated with DHA alone or in combination with Nrf2 inhibitor brusatol. The neurological function, cognitive ability, and cerebral edema degree of rats were assessed. The apoptosis rate and viability of cells was measured. The generation of NOX_2_, Nrf2, HO-1 and NQO-1 expression levels, and ROS content in hippocampal CA1 region and hippocampal neurons were detected. DHA could not only improve the neurological function, brain edema and cognitive ability in TBI rats, but also decrease effectively the contents of NOX_2_ and ROS in hippocampal CA1 region and hippocampal neurons. DHA promoted the nuclear transposition of Nrf2 and the expression levels of HO-1 and NQO-1 in hippocampal CA1 region and hippocampal neurons. On the contrary, Nrf2 inhibitor brusatol inhibited the nuclear transposition of Nrf2 and the expression levels of HO-1 and NQO-1 in hippocampal neurons, promoted the generation of ROS and NOX_2_, and accelerated cell apoptosis. Both in vivo and in vitro experiments demonstrated that DHA treated TBI by reducing NOX_2_ generation that might function on Nrf2 signaling pathway, providing a potential evidence for its clinical application.

## Introduction

Traumatic brain injury (TBI) is one of the common critical cases in neurosurgery, which is one of the main causes of death and disability of young people all over the world [1]. The mechanism of TBI includes primary injury and secondary injury, primary injury mainly refers to the damage of neurons, glial cells and blood vessels caused by the mechanical force at the moment of craniocerebral injury [2]. Secondary injury refers to the inflammatory response, microglia activation, and oxidative stress occurring after primary injury, which is the main mechanism of chronic neuronal degeneration and neurological dysfunction after craniocerebral injury [3]. In contrast, the time of secondary injury is relatively long, providing a treatment window. In the course of pathology, oxidative stress is considered as the core link of secondary injury [4]. Reactive oxygen species (ROS) are important substances involved in oxidative stress [5]. There are growing evidences indicate that NADPH oxidase (NOX_2_) is the main source of ROS, and it is widely expressed in nervous system after craniocerebral injury [6]. Moreover, previous findings have shown that NOX_2_ deficiency can reduce oxidative stress, alleviate neuroinflammation, and improve neuron survival and functional outcome after TBI [7]. Therefore, NOX_2_ may be a crucial target for the treatment of TBI.

Docosahexaenoic acid (DHA) is a highly unsaturated fatty acid necessary for human body, which can maintain the normal structure and physiological function of cells [8]. DHA has a certain positive effect on central nervous system, with the advantages of less side effects and strong therapeutic effects, thus it may be a promising drug for the treatment of brain injury [9]. In previous reports, Lin Y et al. [10] demonstrated that DHA could alleviate cerebral ischemia reperfusion injury, maintain the integrity of the blood–brain barrier, and reduce the permeability of the blood–brain barrier in rats. Chen Xiaobo et al. [11] found that DHA played a protective role in cerebral microvascular endothelial cells and astrocytes in rats with oxysugar/sugar deficiency. In the TBI rat models, Zhu W et al. [12] pointed out that DHA prevented neuron death and alleviated TBI injury through regulating apoptosis-related proteins. Moreover, DHA has been verified to provide neuroprotection for the TBI rats by activating nuclear factor erythroid 2-related factor 2 (Nrf2) signaling pathway [13]. Nonetheless, the role of NOX_2_ in neuroprotective effects of DHA has not been noticed, so we conducted the following experiments with this as a starting point.

Here, TBI rat models were constructed and treated with DHA, the neurofunction, NOX_2_ expression in hippocampal CA1 region and correlated Nrf2 pathway by using Nrf2 inhibitor were analyzed. The purpose of this study was to clarify the changes of NOX_2_ in DHA treatment on TBI, so as to further explore the mechanism of DHA treating ITBI, relating to NOX_2_ generation and Nrf2 signaling pathway, to provide a potential evidence for the clinical application of the drug.

## Materials and Methods

### Statement

The animal experiments performed in this study was supported by the Animal Ethical Committee of the Affiliated Yantai Yuhuangding Hospital of Qingdao University, and the experimental procedures conformed the Guidelines of The Use of Laboratory Animals in China.

### Animal Experiment

Male Sprague–Dawley rats (n = 100; age: 10–12 weeks; weight: 250–300 g) involved in this study were provided by Guangzhou Medical Laboratory Animal Center (Guangzhou, China). All rats were raised under controlled conditions (temperature at (21 ± 2) °C; relative humidity of 60–70%; light/dark cycle (12/12 h)), and had free access to water and food without DHA. After 1 week of feeding, the rats were evenly divided into four groups: sham, TBI, TBI + DHA and TBI + vehicle (V). Among them, some rats were sacrificed by overdose anesthesia 3 days after TBI treatment, and the brain and hippocampus were isolated for cerebral edema assessment (n = 5/group) and histological detection (n = 10/group). The remaining surviving rats (n = 10/group) were evaluated for neurological function and water maze tests.

As previously mentioned [14], rats in TBI group were narcotized with 1% pentobarbital (30 mg/kg). Then, the head of the rat was put on a stereotactic frame for fixation. Then, cut through the midline scalp to reveal skull, and the 5 mm craniotomy was implemented on the right parietal cortex, with the region from coronal suture to 2.5 mm lateral to the sagittal suture (depth: 2.5 mm, velocity: 5 m/s, dwell time: 100 ms). Subsequently, the bone flap was rapidly replaced, sealed and sutured. All operations were performed in a sterile environment, and the rectal temperature of rat was kept at 37 °C. For comparison, rats received surgery same to TBI group without TBI injury, were served as sham group. In TBI + DHA and TBI + V groups, rats respectively received DHA (55 mg/kg/day; #D2534, Sigma-Aldrich, St. Louis, MO, USA) or equivalent 0.9% normal saline gavage after TBI surgery for 30 min. In the end, rats were put back into the feeding room after the anesthesia failed.

### Neurological Severity Score (NSS) Test

After treatment of 12 h, 1 day, 3 days, 7 days, 14 days and 21 days, NSS test was used to evaluate the neurological function of rats in four groups (n = 10/group). In brief, rats in every group were requested to accomplish 10 various missions to assess the functions of alertness, balance and motor. According to the failure of missions, the total score was 0–10, among which 0 indicated that all tasks had been completed and 10 indicated that all tasks were failed. The higher the score, the more serious the neurological injure of the rats.

### Cerebral Edema Assessment

The degree of cerebral edema was assessed though the relative cerebral water content analysis with the wet-dry weight method as reported in advance [15]. After 3 days of operation, brains of rat (n = 5) were extracted and immediately weighed to get wet weight, and then dried at 110 °C for 48 h to acquire the dry weight. The relative cerebral water content was calculated as follows: (wet weight—dry weight) / wet weight × 100%.

### Water Maze Test

From the 7th to 10th day after operation, water maze test [16] was used to evaluate the hippocampus-dependent cognitive ability, including spatial learning and memory ability (n = 10/group). Before the operation, all rats were trained to reach the target platform. In each test, the rat was permitted to find the platform within 60 s. On the last day, the target platform was dislodged, and the time the experimental rats spent in target quadrant was recorded. Finally, the average swimming velocity of each group was calculated.

### NOX_2_ Activity

3 days after operation, the hippocampal CA1 region tissues (n = 10) were collected, mixed, and the NOX_2_ activity was measured by the colorimetric method [17]. In brief, hippocampal CA1 region tissues were homogenized (pH 7.4) and centrifuged at 4 °C for 10 min (1000 × *g*), and the supernatants were collected. Then, the supernatants were centrifuged again at 4 °C for 20 min (13,000×*g*) to extract the membrane fractions. 50 μg membrane fractions were applied to evaluate NOX_2_ activity. A luminometer (Shanghai spectrum instrument Co., Ltd, Shanghai, China) was used to measure the relative light units (RLU) for 5 min every minute consecutively.

### Immunohistochemistry Staining

3 days after operation, immunohistochemistry staining was used to detect the content of NOX_2_ in hippocampal CA1 region. For the experiment, hippocampus tissues (n = 10) were respectively routinely fixed with 4% paraformaldehyde solution, dehydrated in graded alcohol, embedded in paraffin, and then sliced into Sects. (5 μm). The samples were sealed with 3% H_2_O_2_ for 20 min to block the activity of endogenous peroxidase, and followed by blocking with 5% goat serum at room temperature for 60 min. Subsequently, the sections were co-cultured with the NOX_2_ primary antibodies (1: 500; ab131083, Abcam, USA) at 4 °C overnight, and then incubated with the homologous horseradish peroxidase (HRP) -cconjugated secondary antibody (1: 2000; ab205718, Abcam, USA) for another 60 min. Then staining color was developed by DAB reagent (Beyotime, Shanghai, China) for 10 min, and the images were observed under the AxioVision4Ac microscope (Carl Zeiss, Germany).

### Cell Experiment

The mouse hippocampal neurons (HT22) were purchased from BeNa Culture Collection (#BNCC337709; Beijing, China), and cultured in Dulbecco’s modified Eagle’s medium (DMEM, Gibco, USA) supplemented with 10% fetal bovine serum (FBS, HyClone, Logan, UT, USA), penicillin/streptomycin of 50 mg/mL and 2 mmol/L glutamine (Gibco, USA) in a humidified incubator with 5% CO_2_ at 37 °C. When the HT22 cells were fused to approximately 80–90%, they were seeded into 96-well plates and divided into four groups: Control, H_2_O_2_, DHA + H_2_O_2,_ and DHA + H_2_O_2_ + brusatol. Correspondingly, cells in H_2_O_2_ group were induced by H_2_O_2_ (500 μmol/L) [18] for 24 h and acted as models. In DHA + H_2_O_2,_ and DHA + H_2_O_2_ + brusatol groups, H_2_O_2_-induced cells were respectively pre-treated with DHA (20 μmol/L) [19] alone or in combination of DHA with Nrf2 inhibitor brusatol (100 nmol/L; #SML1868, Sigma-Aldrich, St. Louis, MO, USA) [20] for 24 h. For comparison, cells without treatment cultured in ordinary medium were used as Control group.

### Cell Viability

After H_2_O_2_ induction for 12, 24 and 48 h, the viability of HT22 cells (5 × 10^3^ cells/well) were measured by 2-(4,5-dimethylthiazol-2-yl)-2,5-diphenyltetrazolium bromide (MTT) assay. Briefly, 20 μL MTT solution (Sigma-Aldrich, USA) was added into cells at corresponding time point and co-incubated for another 2 h at 37 °C. Then, the optical density (OD) of each well was read by the ELX-800 Biotek plate reader (Winooski, USA) at 595 nm.

### Cell Apoptosis

After H_2_O_2_ induction, the apoptotic rates of HT22 cells in four groups were determined by a flow cytometry. The primary procedures were conducted as follows: H_2_O_2_-induced cells were collected and stabilized in 70% ice-cold ethanol for 60 min. Then, cells were respectively stained with 10 μL Annexin V and 5 μL propidium iodide (PI), and the apoptotic rates were analyzed by a FACSCalibur flow cytometer (BD Biosciences, USA) according to the manufacturer’s instructions.

### ROS Content Detection

The contents of ROS in both HT22 cells and rat hippocampus were estimated by an oxidation-sensitive fluorescent probe (DCFH-DA) with the ROS Assay Kit (E004, Nanjing Jiancheng Bioengineering Institute, Nanjing, China). After treatment, cells and tissues were collected, and incubated with 10 μmol/L DCFH-DA for 20 min at 37 °C. The mean fluorescence intensity of ROS level in cells and tissues were detected using a FACSCalibur flow cytometer (BD Biosciences, USA).

### Western Blot (WB) Analysis

For the analysis, the total proteins of mixed hippocampal tissues (n = 10) and HT22 cells were lysed by RIPA buffer (Beyotime Institute of Biotechnology, Jiangsu, China), and the proteins from cytoplasmic and nuclear fractions were separated using the protein extraction kit (Beyotime Institute of Biotechnology) following the manufacturer's descriptions. Bicinchoninic Protein Assay kit (BCA, Pierce, Rockford, IL, USA) was used to measure the protein concentration. Protein samples (40 µg) were loaded on 10% sodium dodecyl sulfate–polyacrylamide gel electrophoresis (SDS-PAGE, Beyotime, Shanghai, China), and then transferred onto polyvinylidene fluoride (PVDF) membranes (Millipore, Billerica, MA, USA). Afterwards, the membranes were blocked with 5% non-fat dried milk for 1 h at room temperature, and exposed to primary antibodies (Abcam, USA) overnight at 4˚C, including Nrf2 (1:1000, ab32518), Heme Oxygenase-1 (HO-1) (1:2000, ab13243), NADPH quinineoxidoreductase-1 (NQO-1) (1:1000, ab28947), NOX_2_ (1:5000, ab129068). Using β-actin (1:1000, ab8226) or Lamin B1 (1:1000, ab220797) as the internal reference. Subsequently, the homologous secondary antibodies (goat anti-rabbit IgG H&L (HRP; 1:7000, ab97051) and goat anti-mouse IgG H&L (HRP; 1:1000, ab150113)) were added at room temperature for another 1 h. The blots were developed with the enhanced chemiluminescence -detecting kit (ECL; Thermo Fisher, USA).

### Quantitative Real-Time Polymerase Chain Reaction (qRT-PCR) Assay

Total RNA of tissues and cells was isolated using TRIzol reagent (Invitrogen, Carlsbad, California, USA). The quality and integrity of total RNA were detected by the NanoDrop-2000c spectrophotometer (Thermo Fisher Scientific, Massachusetts, USA) and 1% agarose modified gel electrophoresis, respectively. The first-strand cDNA was synthesized from the isolated RNA (1 µg) with the PrimeScript RT Master Mix Perfect Real Time (TaKaRa, Shiga, Japan) in line with the manufacturer’s instructions. qRT-PCR assay was implemented by the ABI Prism 7500 Fast Real-time PCR System (Applied Biosystems, Foster City, CA), and the reaction conditions were set as follows: 40 cycles of 10 min at 50 °C, 10 min at 95 °C, 15 s at 95 °C and 45 s at 45 °C. The corresponding mRNA expression levels were normalized to β-actin, and the data were evaluated by the comparative 2^−ΔΔCt^ method [21]. The sequences of primers were shpwed in Table 1 and synthesized by Gene Pharma (Shanghai, China).

**Table 1:** Primer base sequence

Gene		Forward (5′–3′)	Reverse (5′–3′)
HO-1	Mouse:	GATAGAGCGCAACAAGCAGAA	CAGTGAGGCCCATACCAGAAG
	Rat:	CACGCATATACCCGCTACCT	AAGGCGGTCTTAGCCTCTTC
NQO-1	Mouse:	GGATTGGACCGAGCTGGAA	AATTGCAGTGAAGATGAAGGCAAC
	Rat:	GTGGTGATGGAAAGCAAGGT	GCCCGGATATTGTAGCTGAA
NOX_2_	Mouse:	CCCTTTGGTACAGCCAGTGAAGAT	CAATCCCAGCTCCCACTAACAT
	Rat:	TTGTTGCAGGAGTGCTCATC	CTGCCAGCAGGTAGATCACA
β-actin	Mouse:	TCTTCCAGCCCTCCTTCCTG	AACAGTCCGCCTAGAAGCAC
	Rat:	CCCATCTATGAGGGTTACGC	TTTAATGTCACGCACGATTTC

### Statistical Analysis

Statistical Package of the Social Sciences 20.0 software (SPSS, Inc., Chicago, USA) was used for data analysis. The measurement data were presented as mean ± standard deviation (SD). The difference between groups was performed by Student's *t*-test or one-way analysis of variance (ANOVA) followed by Turkey’s *t*-test. All experiments in vivo and in vitro were performed in triplicate. *P* < 0.05 was considered as statistically significant.

## Results

### DHA Improved Neurological Function and Hippocampus-Dependent Cognitive Ability in TBI Rats, and Reduced Cerebral Edema

**Fig. 1:**
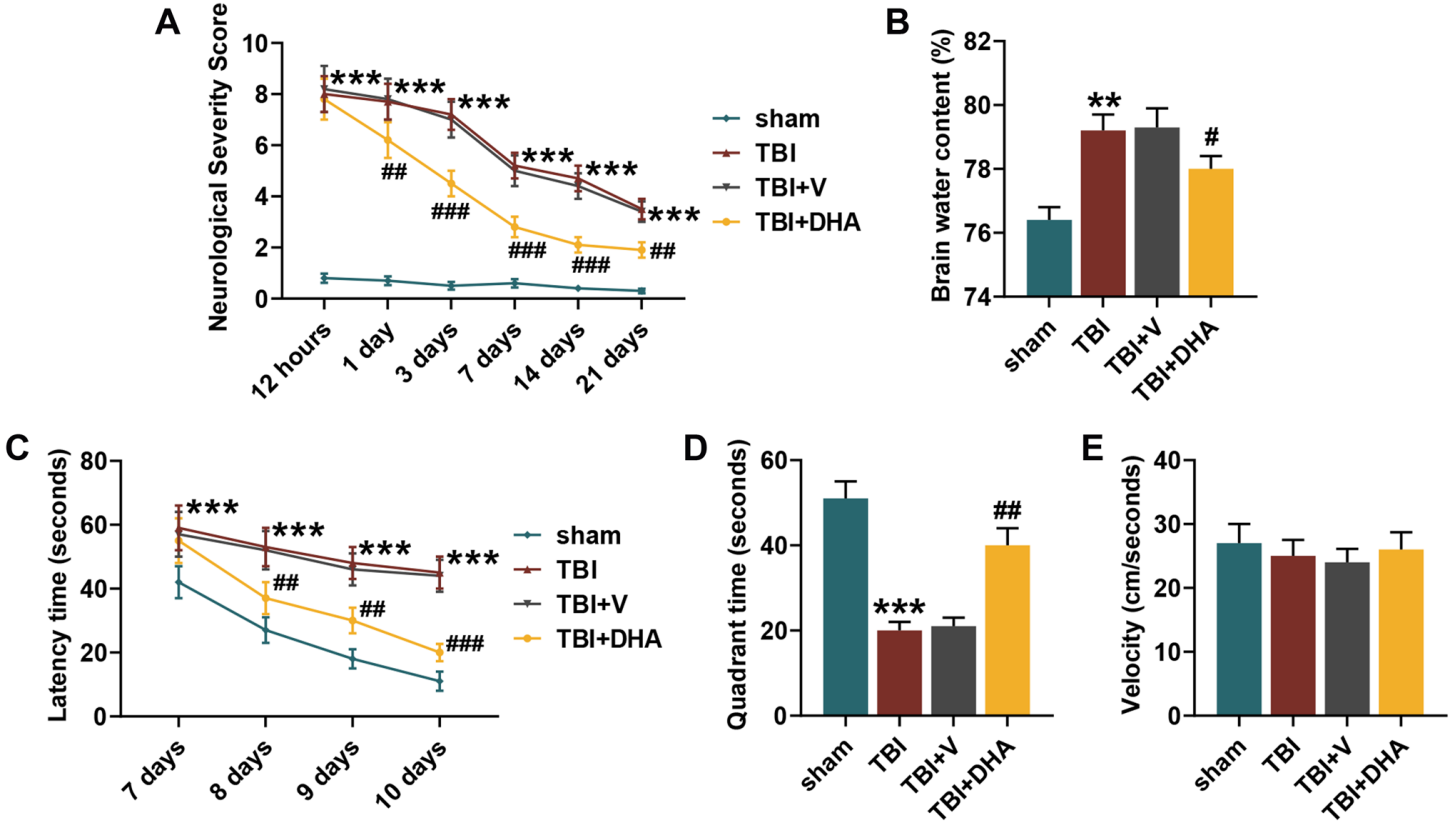
Docosahexaenoic acid (DHA) improved neurological function and hippocampus-dependent cognitive ability in traumatic brain injury (TBI) rats. In this figure, a total of 100 rats were evenly divided into four groups: sham, TBI, TBI + DHA and TBI + vehicle (V). **a** After treatment of 12 h, 1 day, 3 days, 7 days, 14 days and 21 days, neurological severity score (NSS) was used to evaluate the neurological function of rats in four groups. **b** The degree of cerebral edema in four groups was assessed though the relative cerebral water content analysis with the wet-dry weight method. **c**–**e** Water maze test was performed to ascertain the hippocampus-dependent cognitive ability, including latency time, quadrant time and velocity. ***P < 0.001, **P < 0.01, vs. sham; ^#^*P* < 0.05, ^##^*P* < 0.01, ^###^*P* < 0.001, vs. TBI + V

After modeling, we evaluated the neurological function of the rats and found that the NSS score of the rats in TBI group was sharply elevated in comparison with sham group, whereas the neurological function damage caused by TBI could be reversed after 1 day of DHA treatment (*P* < 0.01, Fig. 1a). In cerebral edema assessment, it could been seen from Fig. 1b that, compared with sham rats, the brain water content of rats in TBI group was appreciably increased, which could be rescued by DHA treatment (*P* < 0.05). On 7–10 days after the treatment, the water maze test was conducted to appraise the hippocampal dependent cognitive ability of rats. As shown in Fig. 1c, in contrast to sham group, the latency time of rats in TBI group obviously prolonged, which was effectively shortened after 1 day of DHA treatment (*P* < 0.01). On 10th day, after removal of the target platform, the time rats of TBI group spent in the corresponding quadrant was significantly reduced, while DHA helped increase the quadrant time (*P* < 0.01, Fig. 1d). To ensure comparability, there was no significant difference in the average swimming velocity of rats in the four groups (Fig. 1e).

### DHA Played a Protective Role in TBI Rats Through Nrf2 Signaling Pathway

**Fig. 2:**
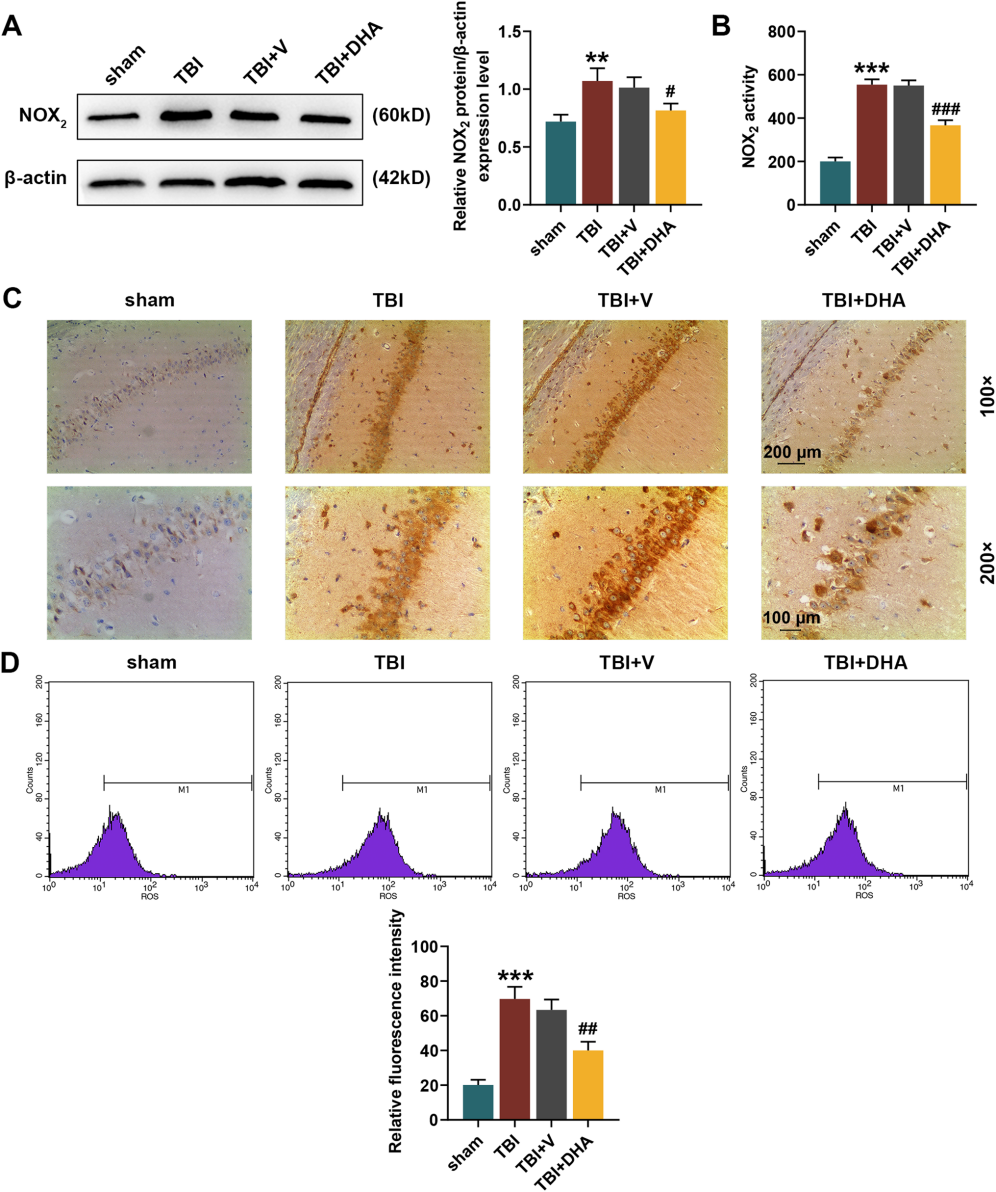
Docosahexaenoic acid (DHA) treatment reduced the NADPH oxidase (NOX_2_) level and reactive oxygen species (ROS) content in hippocampal CA1 region of traumatic brain injury (TBI) rats. In this figure, a total of 100 rats were evenly divided into four groups: sham, TBI, TBI + DHA and TBI + vehicle (V). **a** Western blot (WB) analysis determined the expression level of NOX_2_ in brain tissues of rats in four groups. **b** The colorimetric method was performed to detect the activity of NOX_2_ in hippocampal CA1 region tissues of rats in four groups. **c** Immunohistochemistry staining was used to detect the content of NOX_2_ in hippocampal CA1 region. **d** Flow cytometry analysis determined the ROS content in the hippocampal tissues of rats in four groups. ***P < 0.001, **P < 0.01, vs. sham; ^#^*P* < 0.05, ^##^*P* < 0.01, ^###^*P* < 0.001, vs. TBI + V

**Fig. 3:**
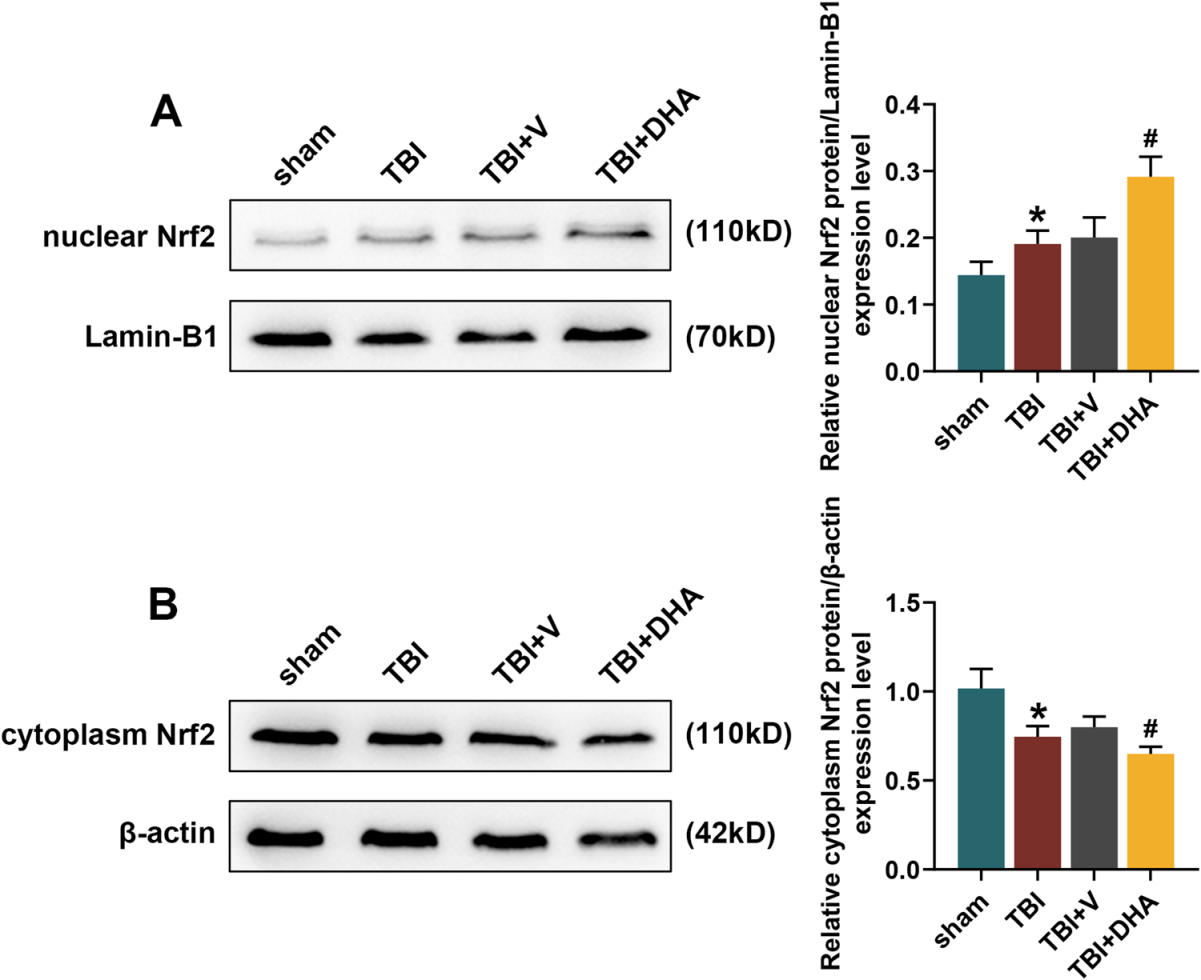
Docosahexaenoic acid (DHA) promoted Nrf2 transport from cytoplasm to nucleus in hippocampal tissues. In this figure, a total of 100 rats were evenly divided into four groups: sham, TBI, TBI + DHA and TBI + vehicle (V). **a**, **b** Western blot (WB) analysis determined the expression level of Nrf2 in nucleus and cytoplasm in hippocampal tissues of rats in four groups. *P < 0.05, vs. sham; ^#^*P* < 0.05, vs. TBI + V

**Fig. 4:**
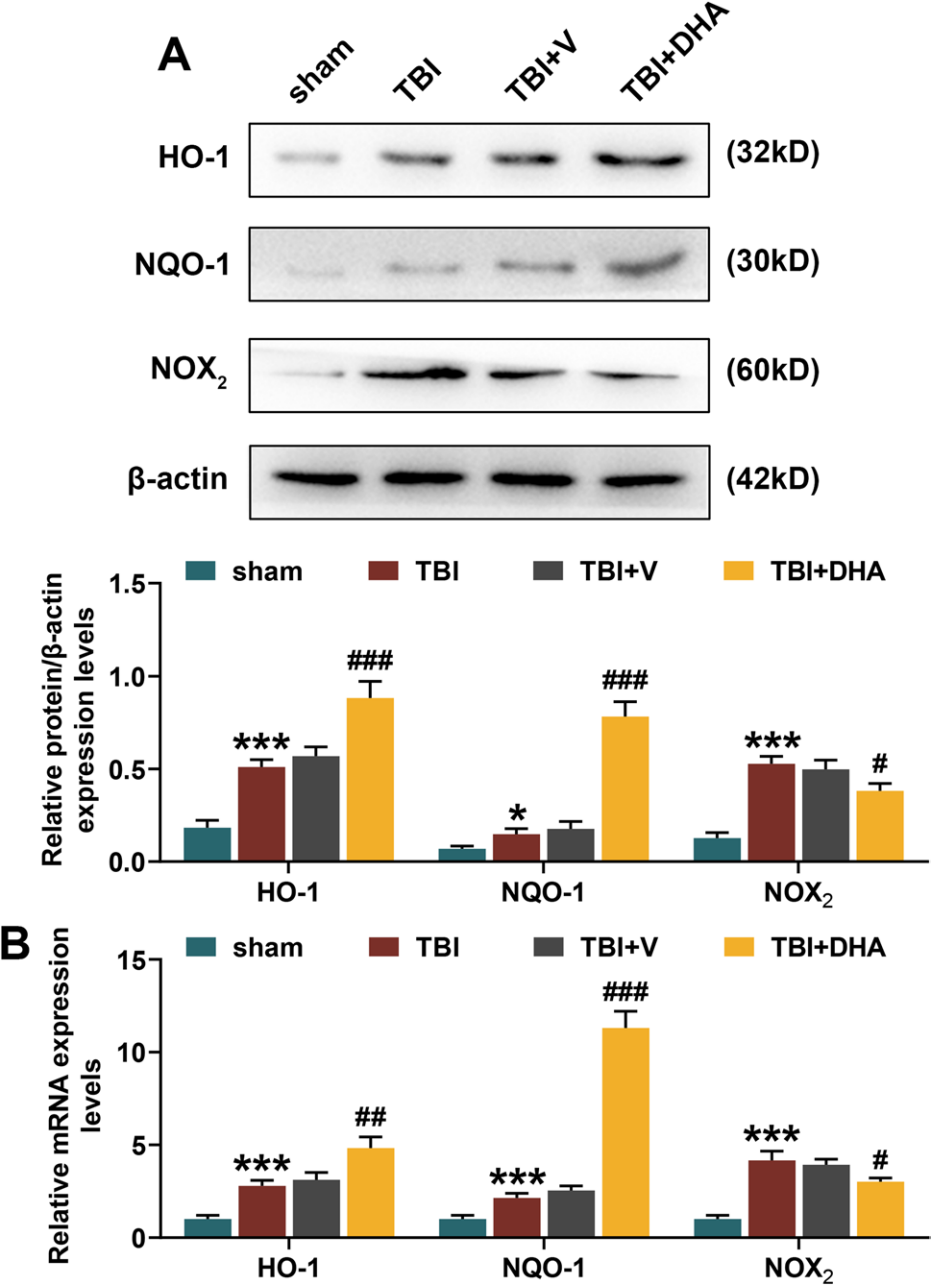
Docosahexaenoic acid (DHA) played a protective role in TBI rats through Nrf2 signaling pathway. In this figure, a total of 100 rats were evenly divided into four groups: sham, TBI, TBI + DHA and TBI + vehicle (V). **a** Western blot (WB) and **b** quantitative real-time polymerase chain reaction (qRT-PCR) assays determined the expression levels of HO-1, NQO-1 and NADPH oxidase (NOX_2_) in hippocampal tissues of rats in four groups. ***P < 0.001, *P < 0.05, vs. sham; ^#^*P* < 0.05, ^##^*P* < 0.01, ^###^*P* < 0.001, vs. TBI + V

WB, colorimetry and immunohistochemistry staining all showed that the level of NOX_2_ was significantly increased in the hippocampal CA1 region of TBI rats, while DHA treatment could reduce the NOX_2_ level in hippocampal CA1 region of TBI rats (*P* < 0.05, Fig. 2a–c). In addition, flow cytometry analysis determined that the ROS content in the hippocampal tissues of TBI rats was distinctly higher than that of sham rats, while DHA treatment could alleviate the increased ROS induced by TBI (*P* < 0.01, Fig. 2d). In the pathway analysis, WB experiment indicated that Nrf2 expression level was increased in the nucleus and decreased in the cytoplasm of hippocampal CA1 region of TBI rats, which was further enhanced by DHA treatment (*P* < 0.05, Fig. 3). Accordingly, both WB and qRT-PCR assays verified that the mRNA and protein levels of HO-1 and NQO-1 were observably raised in hippocampal CA1 region of TBI rats, and their expression levels were further promoted under the treatment of DHA (*P* < 0.05, Fig. 4). Interestingly, DHA inhibited the expression level of NOX_2_ in hippocampal CA1 region of rats induced by TBI (*P* < 0.05, Fig. 4).

### DHA Alleviated H_2_O_2_-Induced Hippocampal Neuron Damage Through Nrf2 Signaling Pathway

**Fig. 5:**
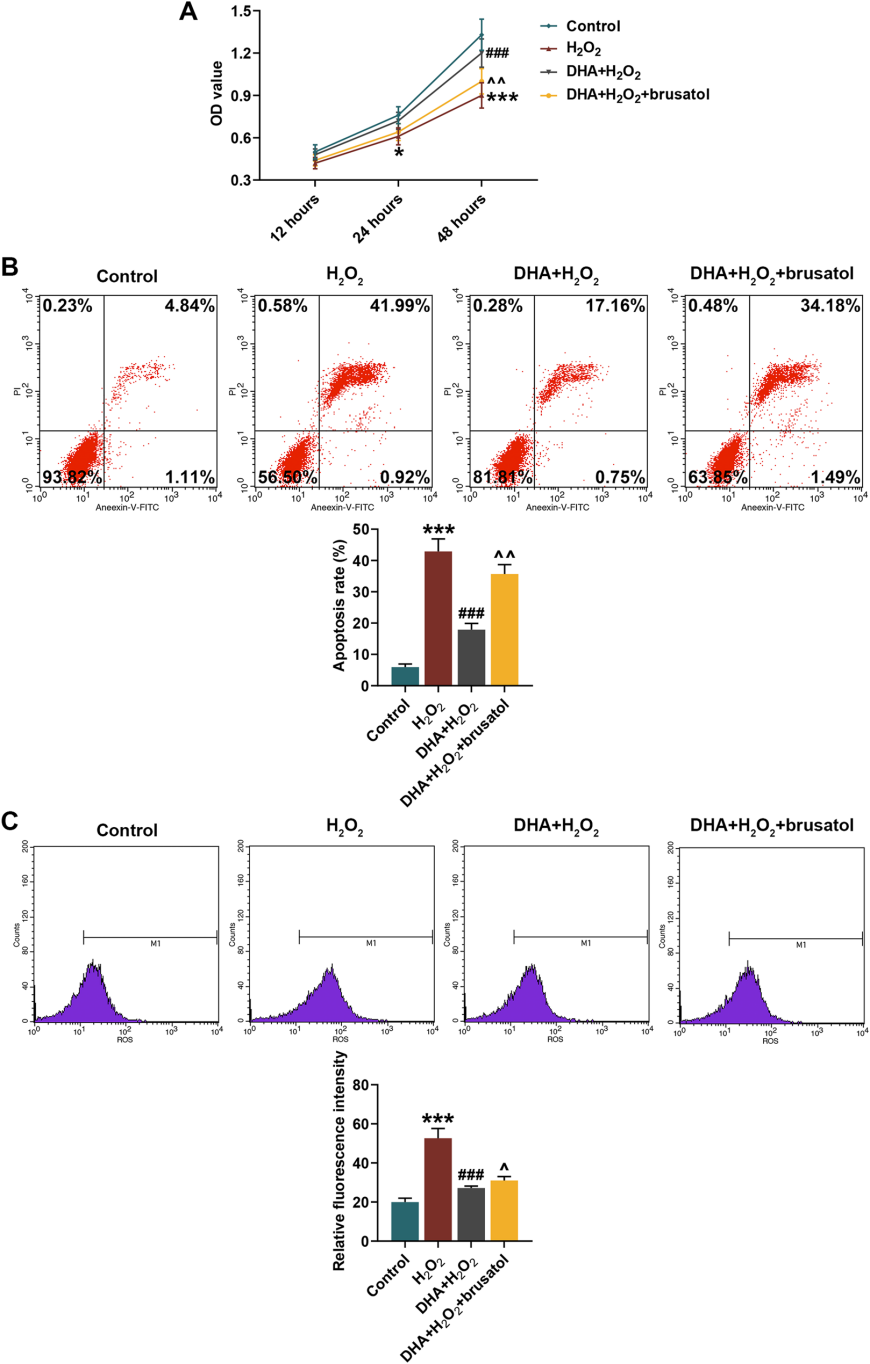
Docosahexaenoic acid (DHA) alleviated H_2_O_2_-induced hippocampal neuron damage. In this figure, mouse hippocampal neurons (HT22) were divided into four groups: Control, H_2_O_2_, DHA + H_2_O_2,_ and DHA + H_2_O_2_ + brusatol. **a** After H_2_O_2_ induction for 12, 24 and 48 h, the viability of HT22 cells in four groups were measured by 2-(4,5-dimethylthiazol-2-yl)-2,5-diphenyltetrazolium bromide (MTT) assay. **b**, **c** Flow cytometry analysis determined the apoptosis rate and reactive oxygen species (ROS) content in HT22 cells after induction. ***P < 0.001, vs. Control; ^###^*P* < 0.001, vs. H_2_O_2_; ^^^*P* < 0.05, ^^^^*P* < 0.01, vs. DHA + H_2_O

**Fig. 6:**
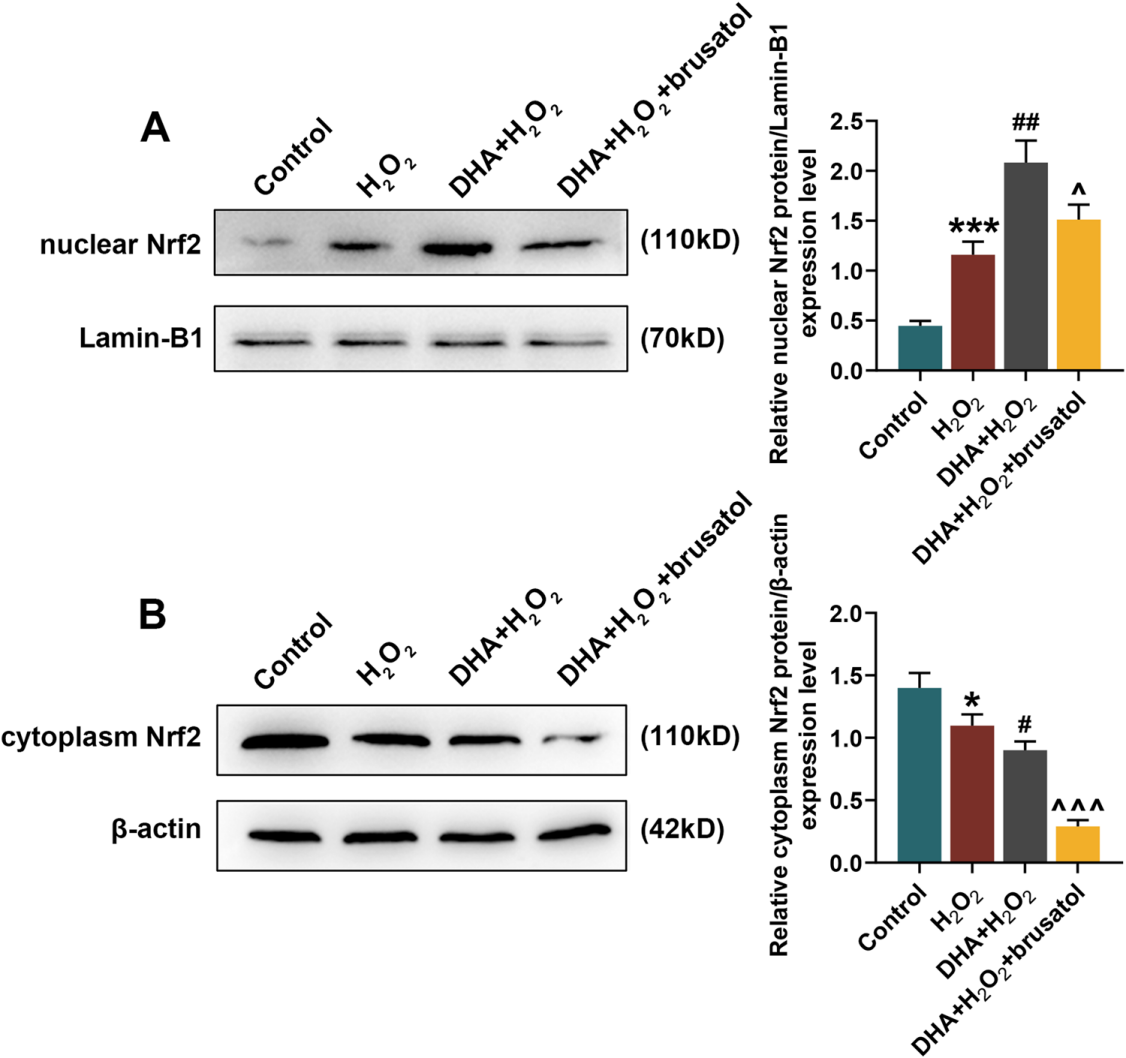
Docosahexaenoic acid (DHA) promoted Nrf2 transport from cytoplasm to nucleus in hippocampal neurons. In this figure, mouse hippocampal neurons (HT22) were divided into four groups: Control, H_2_O_2_, DHA + H_2_O_2,_ and DHA + H_2_O_2_ + brusatol. **a**, **b** Western blot (WB) analysis determined the expression level of Nrf2 in nucleus and cytoplasm in hippocampal neurons of four groups. *P < 0.05, ***P < 0.001, vs. Control; ^#^*P* < 0.05, ^##^*P* < 0.01, vs. H_2_O_2_; ^^^*P* < 0.05, ^^^^^*P* < 0.001, vs. DHA + H_2_O_2_

**Fig. 7:**
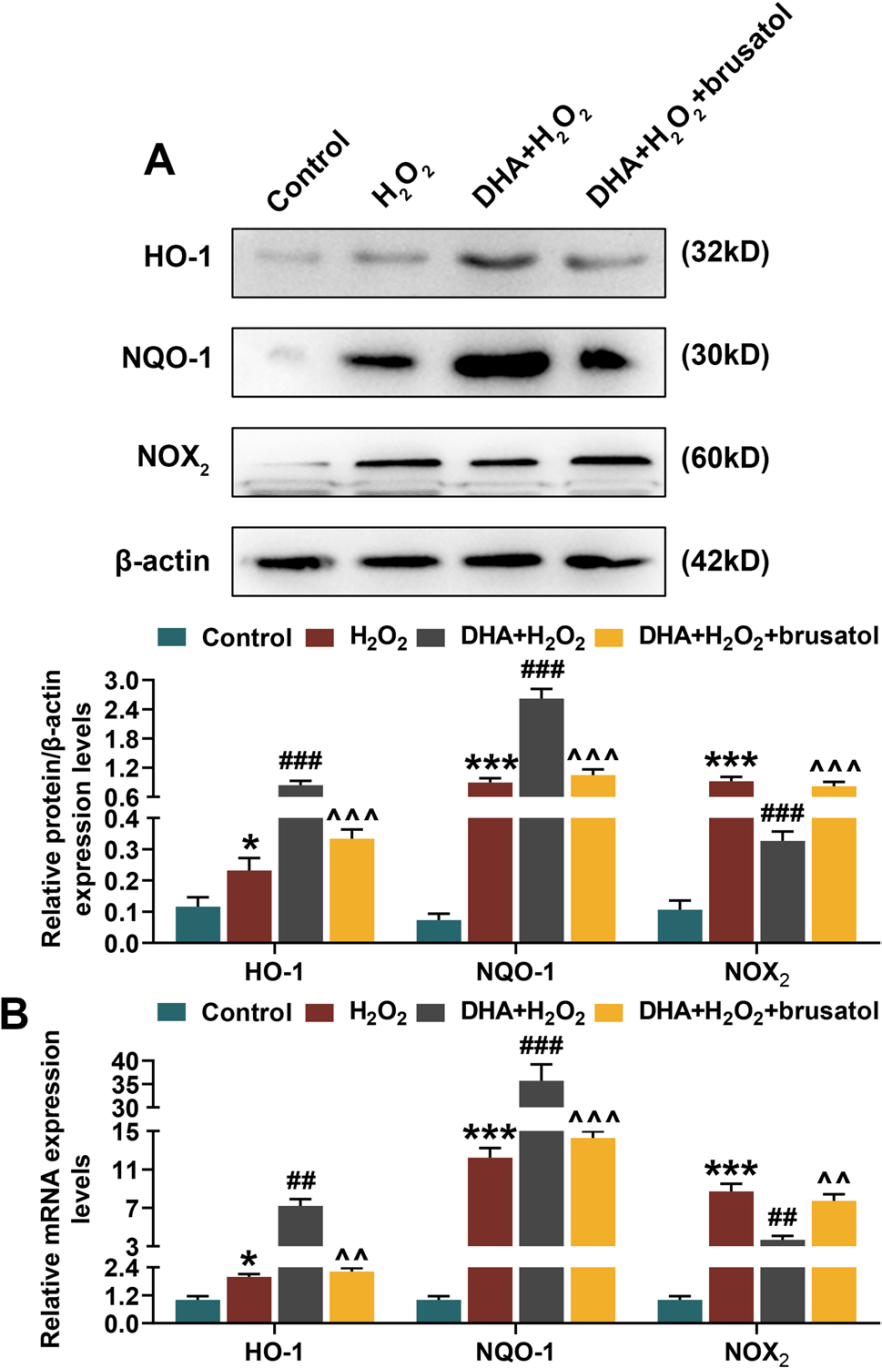
Docosahexaenoic acid (DHA) alleviated H_2_O_2_-induced hippocampal neuron damage through Nrf2 signaling pathway. In this figure, mouse hippocampal neurons (HT22) were divided into four groups: Control, H_2_O_2_, DHA + H_2_O_2,_ and DHA + H_2_O_2_ + brusatol. **a** Western blot (WB) and **b** quantitative real-time polymerase chain reaction (qRT-PCR) assays determined the expression levels of HO-1, NQO-1 and NADPH oxidase (NOX_2_) in hippocampal neuron of four groups. *P < 0.05, ***P < 0.001, vs. Control; ^##^*P* < 0.01, ^###^*P* < 0.001, vs. H_2_O_2_; ^^^^*P* < 0.01, ^^^^^*P* < 0.001, vs. DHA + H_2_O_2_

In cell experiments, DHA helpfully alleviated H_2_O_2_-induced decrease in hippocampal neurons viability, while Nrf2 inhibitor brusatol significantly reversed the protective effect of DHA on hippocampal neurons (*P* < 0.05, Fig. 5a). Consequently, flow cytometry analysis revealed that DHA suppressed the apoptosis and ROS generation in H_2_O_2_-induced hippocampal neurons, which could be reserved by brusatol (*P* < 0.05, Fig. 5b, c). Similar to animal experiments, DHA enhanced Nrf2 expression in nucleus of H_2_O_2_-induced hippocampal neurons and inhibited its expression in cytoplasm, while brusatol reduced Nrf2 expression in both nucleus and cytoplasm (*P* < 0.05, Fig. 6). Moreover, according to the results of WB and qRT-PCR assays, it could be seen that DHA promoted the expression levels of HO-1 and NQO-1 in H_2_O_2_-induced hippocampal neuron, but brusatol reversely decreased the expression of these two (*P* < 0.05, Fig. 7). The production of NOX_2_ in H_2_O_2_-induced hippocampal neuron was restrained by DHA, and could be rescued under the brusatol addition (*P* < 0.01, Fig. 7).

## Discussion

Secondary injury after TBI is the main factor affecting the neurological function of patients, so intervention in the treatment window period is beneficial to improve the prognosis of patients and their long-term quality of life [3]. After TBI injury, NOX_2_ produces excessive ROS, which is the main element of oxidative stress, neurological damage and hippocampus-dependent cognitive dysfunction [22]. In recent years, reducing oxidative stress response has become another direction of treating secondary injury after TBI [23]. With the deepening of the research on the injury mechanism and oxidative stress of TBI, more and more evidences indicate that inhibiting activity of NOX_2_ helps reduce ROS production and delay the progress of the disease [24]. In this study, on the one hand, we found that DHA could not only directly improve the neurological function and reduce brain edema in TBI rats, but also ameliorate the hippocampus-dependent cognitive function. On the other hand, we have confirmed through various experiments that NOX_2_ activity and ROS content in the hippocampal CA1 region of TBI rats could be effectively depressed under the treatment of DHA. These findings suggested that DHA could reduce ROS production by inhibiting NOX_2_ activity, thereby alleviating TBI induced neurological dysfunction.

As an unsaturated fatty acid, DHA has an excellent antioxidant stress effect in vitro and in vivo, and plays a positive role in improving learning and memory abilities [25]. Harvey LD et al. also found that DHA treated TBI rats had decreased degeneration of neurons in the brain and reduced endoplasmic reticulum stress-related inflammation, thereby promoting disease recovery [26]. The report of Yang et al. [27] declared that DHA treatment could relieve oxidative stress response in hepatoma carcinoma cells via Nrf2 signaling pathway. ZgórzyńskaE et al. [28] confirmed that DHA could activate Nrf2 upstream pathways, thus protecting astrocytes from oxidative stress damage. The transcription factor Nrf2 is a pivotal master regulating the activity of antioxidant enzymes with antioxidant response element (ARE) in promoter [29]. In physiological state, the transcription factor Nrf2 exists in the cytoplasm, binds to the kelch-like ECH associated protein 1 (Keapl), and its activity is relatively inhibited [30]. In the downstream targets of Nrf2, HO-1 and NQO-1 act as antioxidants against cell damage caused by oxidative stress and foreign harmful substances [31], prompting that the activation of Nrf2 signaling pathway is helpful to rescue TBI injury.

Numerous findings have expounded that nuclear Nrf2 level and its downstream targets HO-1 and NQO-1 were triggered after TBI injure [32, 33], as same as the results of this study, which mainly due to the transient increase caused by the stress response after brain injury. Interestingly, the application of DHA in TBI rats not only further promoted the nuclear transposition of Nrf2, but also elevated the expression levels of HO-1 and NQO-1 in hippocampal CA1 region, suggesting that DHA could activate Nrf2 signaling pathway and thus playing a protective role in TBI rats. With one accord, Bang HY et al. [34] showed that DHA induced the up-regulation of HO-1 and NQO1, accompanied by Nrf2 translocation to the nucleus, and thus play an antioxidant stress role in human breast epithelial cells. Therefore, the idea that DHA plays an active role in related diseases by activating Nrf2 signaling pathway is valid.

In this present study, we further probed into that the expression level of NOX_2_ in rat hippocampal CA1 region also showed a rising trend under TBI and that DHA treatment reduced this. Previous findings have pointed out that Nrf2 can regulate ROS production through controlling NOX_2_, so as to maintain intracellular redox homeostasis [35]. Deshmukh P et al. claimed that activation of Nrf2 may have a potential therapeutic effect on oxidative stress caused by ROS overdose, especially for neurodegenerative diseases [36]. So it could be speculated that DHA might be beneficial to TBI by suppressing NOX_2_ activity though Nrf2 signaling pathway. To test our hypothesis, mouse hippocampal neurons (HT22) were induced by H_2_O_2_ to trigger oxidative stress response with the pretreatment of DHA or Nrf2 inhibitor brusatol. In our research, corresponding to animal experiments, DHA improved the activity of H_2_O_2_-induced mouse hippocampal neurons as well as inhibited apoptosis and ROS production; on the other hand, DHA enhanced Nrf2 translocation to the nucleus and suppressed NOX_2_ expression while promoting HO-1 and NQO1 expression in H_2_O_2_-induced mouse hippocampal neurons (HT22). On the contrary, Nrf2 inhibitor brusatol inhibited the activation of Nrf2 signaling pathway, promoted the generation of ROS and NOX_2_, and thus accelerating cell apoptosis and reversing the protective effect of DHA on H_2_O_2_-induced mouse hippocampal neurons. These findings verified that inhibition of Nrf2 signaling pathway could reduce NOX_2_ production even under DHA treatment, thereby promoting oxidative stress response and hippocampal neuron apoptosis. From our research, the findings in H_2_O_2_ injured mouse hippocampal neurons were consistent with that in TBI rat models, that the injury could be reversed by DHA relating to NOX_2_, HO-1 and Nrf2 pathway.

It might be a limitation not using a NOX_2_ inhibitor to show a reduction in ROS post injury in vivo model, due to the animal ethics.

In conclusion, both in vivo and in vitro experiments have demonstrated that DHA could reduce NOX_2_ generation by inhibiting the activation of Nrf2 signaling pathway, thereby reducing hippocampal neuron apoptosis, and improving neurological function and hippocampus-dependent cognitive ability in TBI rats. The experimental results of this study further clarified the function of NOX_2_ in the neuroprotective effect of DHA on TBI, which clarified the mechanism of DHA and provided potential evidences for its clinical application.
